# Q fever infection in dairy cattle herds: increased risk with high wind speed and low precipitation

**DOI:** 10.1017/S0950268814003926

**Published:** 2015-03-18

**Authors:** S. NUSINOVICI, J. FRÖSSLING, S. WIDGREN, F. BEAUDEAU, A. LINDBERG

**Affiliations:** 1 INRA, UMR1300 Biology, Epidemiology and Risk Analysis in Animal Health, CS 40706, F-44307 Nantes, France; 2 LUNAM Université, Oniris, UMR BioEpAR, CS 40706, F-44307 Nantes, France; 3 National Veterinary Institute, Travvägen, Uppsala, Sweden

**Keywords:** Control measure, precipitation, Q fever, risk factor, wind

## Abstract

Ruminants are considered the main reservoir for transmission of *Coxiella burnetii* (*Cb*) to humans. The implementation of effective control measures against *Cb* in ruminants requires knowledge about potential risk factors. The objectives of this study were (i) to describe the spatial distribution of Q fever-infected dairy cattle herds in Sweden, (ii) to quantify the respective contributions of wind and animal movements on the risk of infection, while accounting for other sources of variation, and (iii) to investigate the possible protective effect of precipitation. A total of 1537 bulk milk samples were collected and tested for presence of *Cb* antibodies. The prevalence of test-positive herds was higher in the south of Sweden. For herds located in areas with high wind speed, open landscape, high animal densities and high temperature, the risk of being infected reached very high values. Because these factors are difficult to control, vaccination could be an appropriate control measure in these areas. Finally, the cumulated precipitation over 1 year was identified as a protective factor.

## INTRODUCTION

*Coxiella burnetii* (*Cb*) is the infectious agent responsible for Q fever, a zoonosis with worldwide distribution, except in New Zealand [1]. Infection in humans is usually asymptomatic but can induce acute or chronic disease [2]. In livestock *Cb* infection can lead to abortion, stillbirth and fertility disorders [3]. *Cb* is shed through birth products, faeces, urine, milk and vaginal mucus [4–6]. *Cb* infection in humans and livestock occurs mainly after inhalation of contaminated aerosols, with shedding ruminants being considered as the main reservoir for transmission to humans. Furthermore, *Cb* can exist for up to 150 days in soils [7] and is highly resistant to chemical disinfectants [8]. Environment thus represents another source of contamination.

In this context, the control of the propagation of *Cb* within and between ruminant herds is both an important public health and an animal health issue. The implementation of effective control measures in the ruminant populations should consequently have positive consequences on human health.

Control of *Cb* is driven by current knowledge and experts' opinions on the agent's characteristics and transmission pathways. Within infected herds, possible actions include hygienic measures and medical measures, especially vaccination using a phase I vaccine [9]. At the regional level, control of *Cb* infection relies not only on measures in infected herds, but also on preventive actions that may be implemented in herds still free from infection.

Several studies suggest the role of wind in the transmission of the bacteria between ruminants and humans [10–12]. Moreover, introduction of new animals into cattle herds has been identified as a risk factor of *Cb* infection [13] and it is known that trade between cattle herds occurs frequently and sometimes over long distances [14]. Therefore, it can be assumed that the propagation of the bacteria between ruminant herds may either result from its passive transport through wind and/or the introduction of infected shedder animals into *Cb*-free herds. However, the impact on *Cb* spread between farms of the airborne dispersion relative to movements of possibly infected domestic cattle has not been yet quantified. This information would help to make relevant decisions on which control measures to implement against *Cb* spread. Our assumptions are the following: the passive transport of *Cb* by wind is regarded as uncontrollable; therefore vaccination may be the only adequate control measure to implement, even in *Cb*-free herds, if this measure is to have a major impact on the spread of the pathogen. Alternatively, non-medical measures, such as laboratory tests before purchase in order to detect putative shedders and/or restricted movements between herds having the same *Cb* infection status could be effective in limiting its spread between herds.

The transmission of the bacteria within and between herds and to humans could also be influenced by other factors. First, a higher animal density could increase the risk of propagation by increasing the potential number of neighbouring sources of contamination. This has been shown in cattle herds [13] and between goat farms and humans [12]. Second, open landscapes such as fields could emphasize the risk of airborne transmission because such areas are likely to be windier compared to closed landscapes (e.g. land with forest or buildings). Third, it is known that the bacteria are aerosolized in the environment after shedding and are then carried within inhalable airborne dust [15, 16]. Thus, a higher quantity of precipitation could decrease the quantity of dust in the air and thus the likelihood of transmission. Finally, the survival of the bacteria in the environment may be influenced by temperature.

The objectives of this study were (i) to describe the spatial distribution of *Cb*-infected dairy cattle herds in Sweden, (ii) to quantify the respective contributions of wind and animal movements on the risk of a herd to become infected, while accounting for other sources of variation (type of landscape, local animal densities and temperature), and (iii) to investigate the possible protective effect of precipitation on the risk of infection.

## MATERIALS AND METHODS

### Available data

A systematic random sample was drawn from bulk tank milk (BTM) samples originally submitted for bovine virus diarrhoea virus (BVDV) surveillance, in a scheme that covers > 95% of all Swedish dairy cattle herds. A sampling fraction of 25% was used and the presence of antibodies (ELISA) against *Cb* was investigated using the IDEXX Chekit^®^ Q Fever Antibody kit (IDEXX Laboratories, USA). A total of 1537 samples were collected in October 2008 (*n* = 970) and June 2009 (*n* = 567). Herds included in this study were tested once.

Wind, precipitation and temperature data were obtained from the European Centre for Medium-Range Weather Forecasts [17]. This centre compiles raw data from various sources and reanalyses the data using atmospheric models. The wind-related data consisted of East and North wind vector components, named U and V, respectively, and is expressed in metres per second (m/s). Monthly means of U and V components were extracted. Regarding the precipitation and temperature data, the quantity cumulated per month, expressed in metres and degrees, respectively, were extracted. These data were extracted for the whole country at a spatial resolution of 0·25° × 0·25° latitude and longitude.

Data related to landscape characteristics were obtained from the national authority for geographical information in Sweden [18]. The raw data consisted of the type of landscape at a spatial resolution of 100 m × 100 m with the following categories: water surface, forest, field, other open land, clearing, fruit plantation, bare mountain, low mountain forest, closed settlement, high settlement, low settlement, industrial area, recreation settlement, other open land without forest contour, water surface with diffuse beach line and deciduous forest.

The animal movement data consisted of all commercial transfers from 2005 to 2011. The geographical coordinates of all the cattle and sheep herds were available for analysis. To calculate the local cattle and sheep densities, the number of cattle and sheep that were present in all herds (not only the tested ones) the year before the ELISA test was considered. While goats can contribute to *Cb* spread, they were not considered in this study due to their very small numbers in Sweden [19].

### Construction of variables

The status of each herd with regard to *Cb* infection was determined using antibody detection in the BTM. A herd was considered positive when the S/P ratio was > 40. At this threshold, the sensitivity and specificity of the test was 98·6% and 97·1% for individual milk samples [20].

#### Meteorological data

The meteorological data (wind speed, precipitation, temperature) were aggregated to reflect the year before the ELISA test. To cover this period both for herds tested for antibodies in the last week of October 2008 and in the first week of June 2009, meteorological data between November 2007 and May 2009 were extracted. For wind data, monthly values were averaged over the year before the test (from November 2007 to October 2008 for herds tested in 2008 and from June 2008 to May 2009 for herds tested in 2009). The corresponding U and V components were then combined to calculate the yearly average wind speed using the following equation (1):
1
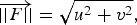

Monthly means and annual averages of climatic data are commonly used in climatic sciences [21, 22]. For precipitation and temperature, data were accumulated over the whole year before the test. For wind speed, precipitation and temperature, the value considered for each herd was the value corresponding to the location of the herd in the grid.

#### Landscape data

It was decided for each landscape category whether or not it corresponded to an open landscape. The following categories were considered as open landscape: water surface, field, other open land, clearing, bare mountain, other open land without forest contour and water surface with diffuse beach line. Then, the percentage of cells (100 m x 100 m) with open landscape was calculated over 25 km^2^ (5 km x 5 km cells). The value considered for each herd was the value corresponding to the location of the herd in the grid.

#### Animal densities

Local cattle and sheep densities were calculated in a 5 km radius around each sampled herd. For the cattle density, both beef and dairy herds were considered because they could both contribute to *Cb* spread. The density was calculated at the animal level because the herd size could influence the risk of infection. For instance, a herd could have a higher risk of infection if located close to a large infected herd compared to a small infected one.

#### Animal movements

Network analysis was used to investigate the impact of animal movement (all commercial transfers, not only transfers from sampled herds). Of network parameters, the in-degree (ID) was considered; it corresponds to the number of contacts with direction to the herd, in our case, the number of herds from which each herd receives animals directly [23]. This network parameter has been used in several studies to investigate animal movements in the context of disease control and risk-based surveillance [24–26]. The ID parameter was calculated over a 1-year time period before the result of the ELISA test.

### Statistical analysis

#### Spatial analysis

Putative clusters of herds positive by the ELISA test on BTM were detected by spatial statistical analysis using a Bernoulli model in SaTScan [27, 28]. The herds' coordinates were considered as points (rather than aggregated information). The maximum spatial cluster size used corresponded to 10% of the population at risk. The window shape used allowed the detection of both circular and elliptic areas.

#### Hierarchical clustering

**Fig. 1.**
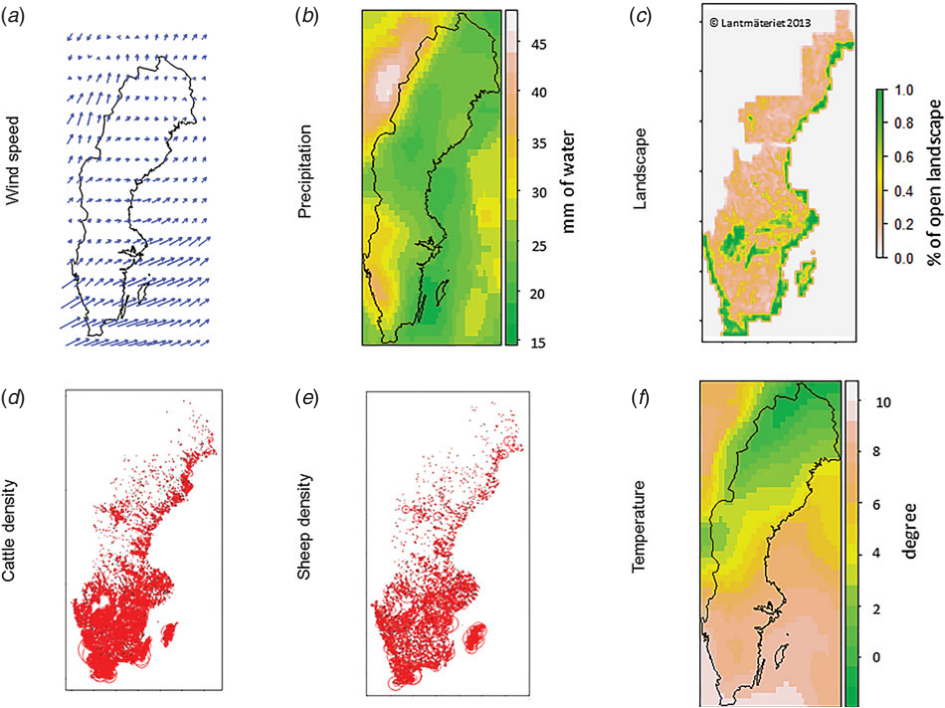
Spatial description of the climatic and environmental variables considered as possible risk factors for *Coxiella burnetii* infection. (*a*) Wind-related data is an average of data from the year before the ELISA test (Q1 = 1·04, median = 1·42, Q3 = 1·70). The angles of the arrows correspond to the wind direction and the lengths of arrows to the wind speed (m/s). The spatial resolution chosen for this figure (1° x 1° latitude and longitude) does not correspond to the resolution used for the analysis (0·25° x 0·25°). (*b*) Total cumulative precipitation in millimetres of water, for the year before the ELISA test (Q1 = 22·0, median = 25·1, Q3 = 29·2). (*c*) Percentage of open landscape calculated over a 5 km x 5 km area (Q1 = 0·28, median = 0·38, Q3 = 0·59). (*d*) Cattle density calculated within a 5 km radius around each herd included in the study (*n* = 1537) considering both dairy and beef cattle (Q1 = 3·8, median = 7·3, Q3 = 13·0). The length of the radius is proportional to the animal density. (*e*) Sheep density calculated within a 5 km radius around each herd included in the study (*n* = 1537) (Q1 = 1·1, median = 2·4, Q3 = 4·3) The length of the radius is proportional to the animal density. (*f*) Average temperature calculated the year before the ELISA test (Q1 = 6·8, median = 7·7, Q3 = 8·3); Sweden, November 2007–May 2009.

**Fig. 2.**
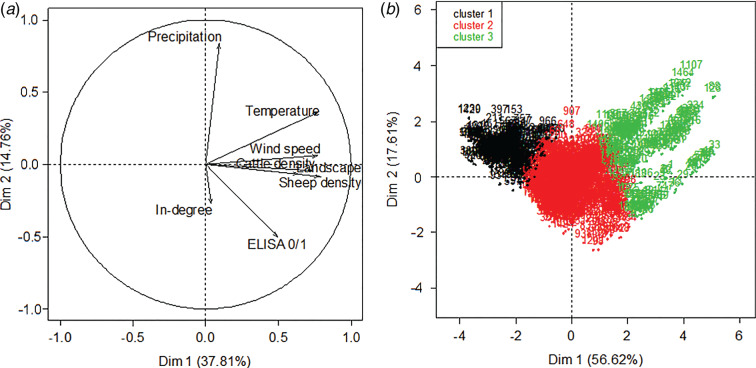
(*a*) Principal component analysis (PCA). This analysis was performed on the following variables: result of the ELISA test against *Coxiella burnetii* (ELISA 0/1), wind speed, cumulated precipitation, percentage of open landscape (Landscape), animal movements (In-degree), cattle and sheep densities and temperature. (*b*) Hierarchical clustering performed on the first two components of the PCA [using the five correlated variables wind speed, percent of open landscape, animal densities (cattle and sheep) and temperature]. In all, 1537 Swedish dairy herds tested in 2008–2009 were included in this study on risk factors for *C. burnetii* infection.

Wind data, percent of open landscape, animal densities (cattle and sheep) and temperature were strongly correlated (Figs 1 and 2*a*). To avoid multicollinearity issues while still keeping the information contained in these variables, it was decided to aggregate them into a single variable to be used in the multivariable model. To do so, a principal component analysis (PCA) was performed using the correlated variables, as proposed by Dohoo *et al*. [29], and then a hierarchical clustering was performed on the first two components of the PCA (Fig. 2*b*). This method allows the identification of different groups based on the distance between herds in the two-dimensional PCA projections [30].

#### Risk factor analysis

The risk for a herd to be detected as BTM ELISA positive in relation to the three independent variables was assessed using logistic regression, as described by equation (2):
2
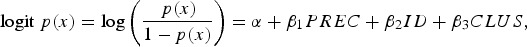

where *p*(*x*) is the risk for a herd to be detected as ELISA positive for BTM, *α* is the intercept, *PREC* is the precipitation variable (four classes), *ID* is the in-degree parameter variable (three classes) and *CLUS* is the hierarchical cluster variable (three classes). *β*_1_, *β*_2_ and *β*_3_ are the adjusted regression coefficients associated to the variables estimated by the model.

#### Analysis of residuals

To check whether the residuals of the logistic regression model were autocorrelated, the variogram quantifying the semivariance between pairs of observations (herd residuals) as a function of their Euclidean distance was plotted. Envelopes for the variogram were based on 999 Monte Carlo permutations of the data, whereby positive and negative herds were randomly allocated to each farm location. Because of this random allocation, a distribution that falls within the envelopes implies that the residuals are not more correlated than what would be obtained by chance.

#### Software

Logistic regressions were performed using R software [31]. In-degree parameters were calculated using the ‘EpiContactTrace’ package in R [32]. PCA and hierarchical clustering were performed using ‘FactoMineR’ [33] packages.

## RESULTS

### Spatial distribution of *Cb*-infected dairy herds

**Fig. 3.**
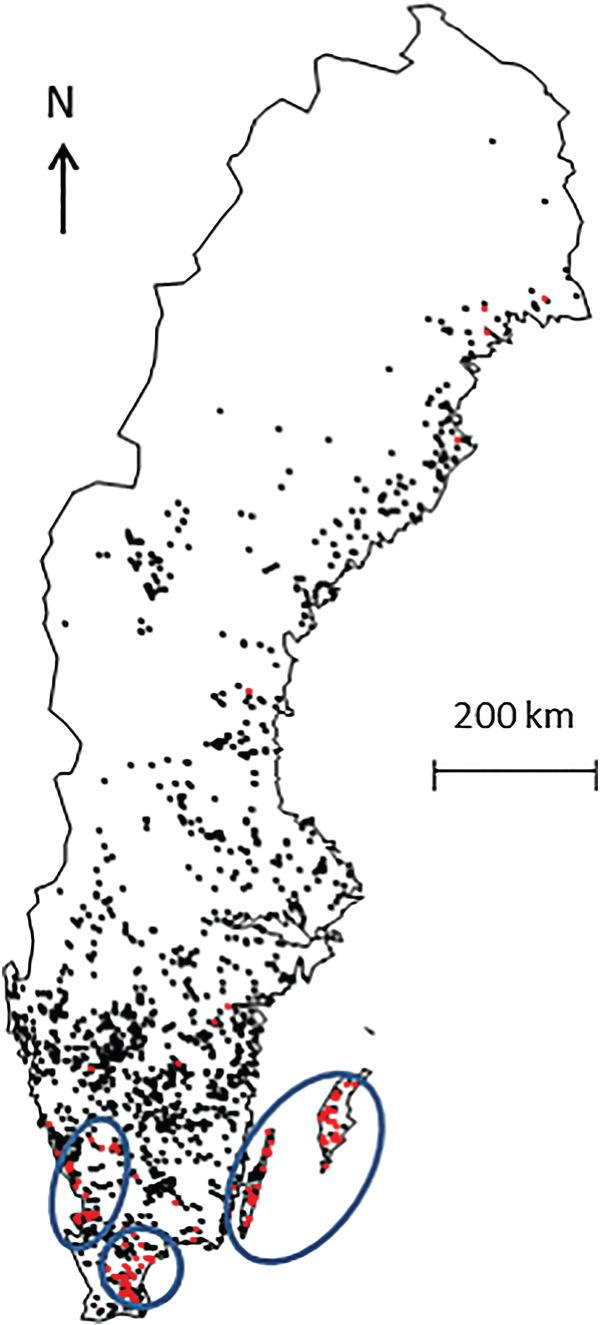
Location of the dairy herds included in the study (*n* = 1537). The red (*n* = 126) and black (*n* = 1411) dots represent herds that were positive and negative to ELISA against *Coxiella burnetii* (*Cb*) in the bulk tank milk, respectively. The blue circles correspond to areas with a higher risk of being detected ELISA positive. Sweden, October 2008–June 2009.

Herd prevalence of antibodies against *Cb* in the BTM in the tested herds was 8·2%. Three clusters associated with an increased risk of being positive to the ELISA test were identified in the southern part of Sweden (relative risk between 5 and 9) (Fig. 3).

### Descriptive results

#### Raw variables

Percentage of open landscape, animal densities and meteorological variables (wind speed, precipitation, temperature) are distributed very heterogeneously along the north–south axis, as shown in Figure 1. Based on descriptive analysis, the southern region is windier, has more rain, has more open land and higher animal densities and is warmer compared to the northern region. The multicollinearity previously mentioned resulted from these distributions. Animal movements, expressed using the in-degree parameter, did not show this spatial pattern and seem more homogeneous over space (result not shown).

#### Cluster variable

**Fig. 4.**
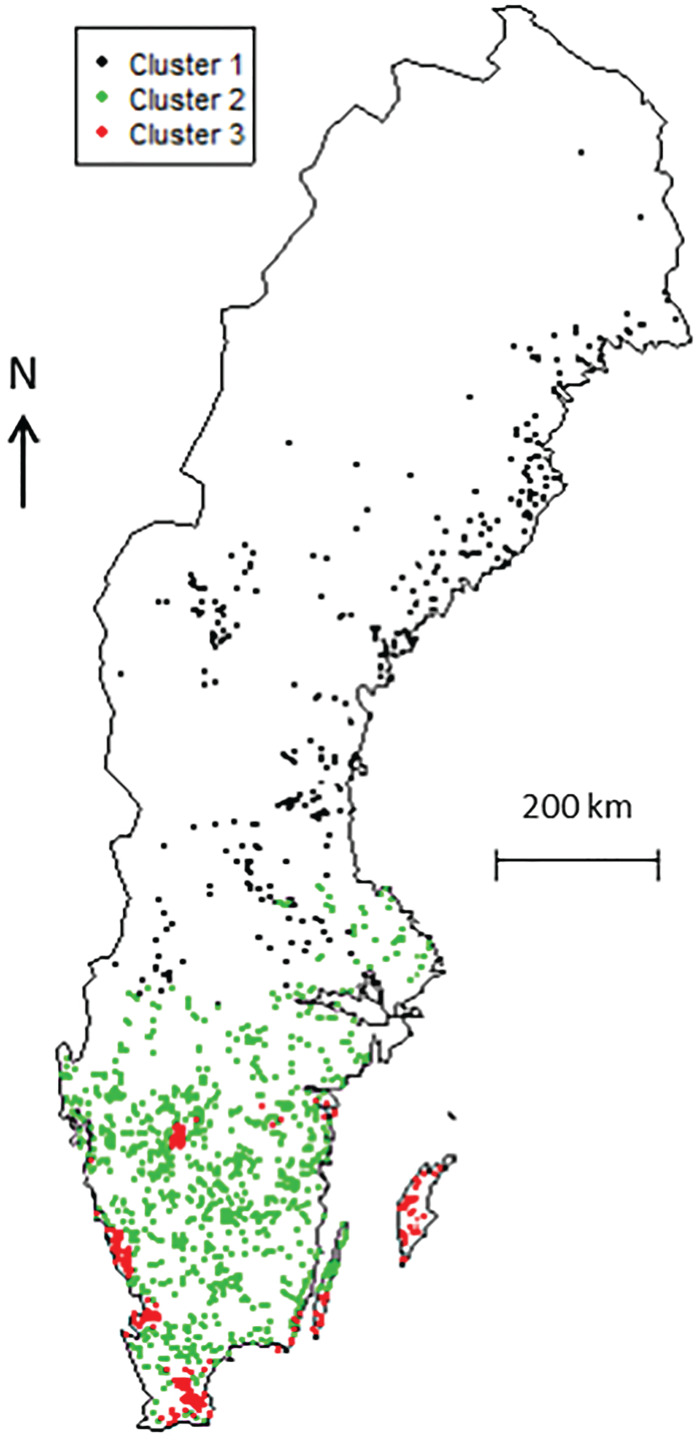
Locations of dairy herds (*n* = 1537) according to the cluster variable. The three clusters resulted from a hierarchical clustering that was performed on the principal component analysis using the following variables: wind speed, percentage of open landscape, animal densities (cattle and sheep) and temperature; Sweden, October 2008–June 2009.

The multicollinearity issue is graphically shown in Figure 2*a* in the PCA. The hierarchical clustering described previously allowed the detection of three clusters of herds (Fig. 2*b*). The distribution of wind speed, percent of open landscape, animal densities and temperature in each cluster are shown in Table 1. Clusters 1 and 3 corresponded to populations with the lowest and highest level of exposure, respectively, while cluster 2 corresponded to an intermediate exposure level. Herds belonging to cluster variable categories 1, 2 and 3 are localized in Figure 4. There was a strong correlation between the ELISA status of the herd and the cluster variable with 4%, 36% and 60% of ELISA-positive herds belonging to clusters 1, 2 and 3, respectively (Cochran–Armitage test, *P* < 0·001).

**Table 1.** Distribution of the wind speed, percentage of open landscape, animal densities (cattle and sheep) and temperature according to the cluster variable

Cluster	No. of herds	Wind speed (m/s)	% of open landscape	Cattle density (cows/km^2^)	Sheep density (sheep/km^2^)	Temperature (°C)
		(Q1, median, Q3)	(Q1, median, Q3)	(Q1, median, Q3)	(Q1, median, Q3)	(Q1, median, Q3)
1	301	0·6, 0·7, 0·9	0·2, 0·3, 0·4	1·0, 2·9, 4·6	0·2, 0·6, 1·3	3·3, 4·1, 4·9
2	1001	1·2, 1·5, 1·7	0·3, 0·4, 0·6	4·5, 7·5, 11·0	1·3, 2·5, 3·8	7·4, 7·7, 8·2
3	236	1·4, 2·1, 2·8	0·6, 0·8, 0·9	17·3, 30·2, 35·4	5·1, 7·7, 9·5	7·9, 8·4, 8·8

The three clusters resulted from a hierarchical clustering that was performed on the principal component analysis using the five variables. In all, 1537 Swedish dairy herds tested in 2008–2009 were included in this study on risk factors for *Coxiella burnetii* infection.

### Risk factor analysis

The results of the bivariate analysis fit with the assumptions. As expected, higher wind speed, animal movements, percent of open landscape, animal densities (both in cattle and sheep) and higher temperatures increased the risk of a herd being ELISA positive. The lower the accumulated precipitation, the higher the risk of infection (Table 2). In the multivariable step, the risk of being ELISA positive in relation to the accumulated precipitation remained in the same range of values. By contrast, the risk of being positive was associated with animal movements and the cluster variable was higher. Interestingly, herds classified in cluster 3 had a 42 times higher risk of being ELISA positive compared to herds classified in cluster 1 (Table 2). The pseudo-*R*^2^ value of this model was 29·2%. Results of the Hosmer–Lemeshow test did not allow for rejection of the null hypothesis that there is no difference between observed and model-predicted values, implying that the model's estimates fit the data at an acceptable level.

**Table 2.** Crude and adjusted odds ratios (OR) with their 95% confidence intervals (CI)

Category*	Crude OR	95% CI	Adjusted OR	95% CI	*P* (Wald's test)
Precipitation (mm)					
[0,22]	Ref.		Ref.		
(22,26]	0·57	0·37–0·88	0·72	0·44–1·18	0·19
(26,31]	0·45	0·28–0·73	0·33	0·19–0·57	<0·001
(31,37]	0·16	0·06–0·41	0·19	0·07–0·51	<0·001
Animal movements					
0	Ref.		Ref.		
[1,3]	1·52	0·96–2·42	1·57	0·97–2·53	0·06
⩾4	2·97	1·87–4·71	3·40	2·01–5·74	<0·001
Wind speed (m/s)					
[0,1]	Ref.				
(1,1·8]	3·53	1·38–11·56			
(1·8,3·3]	19·28	7·77–61·81			
Open landscape (%)					
[0,0·25]	Ref.				
(0·25,0·50]	1·56	0·70–3·85			
(0·50,1]	6·45	3·19–14·77			
Cattle density (cows/km^2^)					
[0,7]	Ref.				
(7,25]	3·08	1·8–5·45			
(25,71]	15·11	8·60–27·41			
Sheep density (sheep/km^2^)					
[0,2]	Ref.				
(2,5]	1·97	1·14–3·44			
(5,16]	6·44	3·88–10·97			
Temperature (°C)					
[0,5]	Ref.				
(5,8]	1·49	0·55–5·04			
(8,10]	11·49	4·65–36·69			
Cluster variable†					
1	Ref.		Ref.		
2	2·79	1·1–9·09	4·23	1·65–10·85	<0·001
3	27·97	11·15–90·44	42·07	16·30–108·5	<0·001

Adjusted ORs were estimated using the output of the logistic regression model. The *P* value calculated using Wald's tests corresponded to the adjusted OR. There are no adjusted results for the wind speed, the percentage of open landscape, animal densities (cattle and sheep) and temperature because they were not included in the multivariable model. All the 1537 dairy herds were included in the bivariate analysis, and 1443 dairy herds that had no missing values were included in the multivariable analysis. The study focused on risk factors for *Coxiella burnetii* infection in Swedish dairy cattle, sampled in 2008–2009.

* Cut-off values used to categorize the continuous variables.

† The cluster variable resulted from the aggregation of the following variables: wind speed, percent of open landscape, animal densities (cattle and sheep) and temperature (see Materials and Methods section for details).

**Fig. 5.**
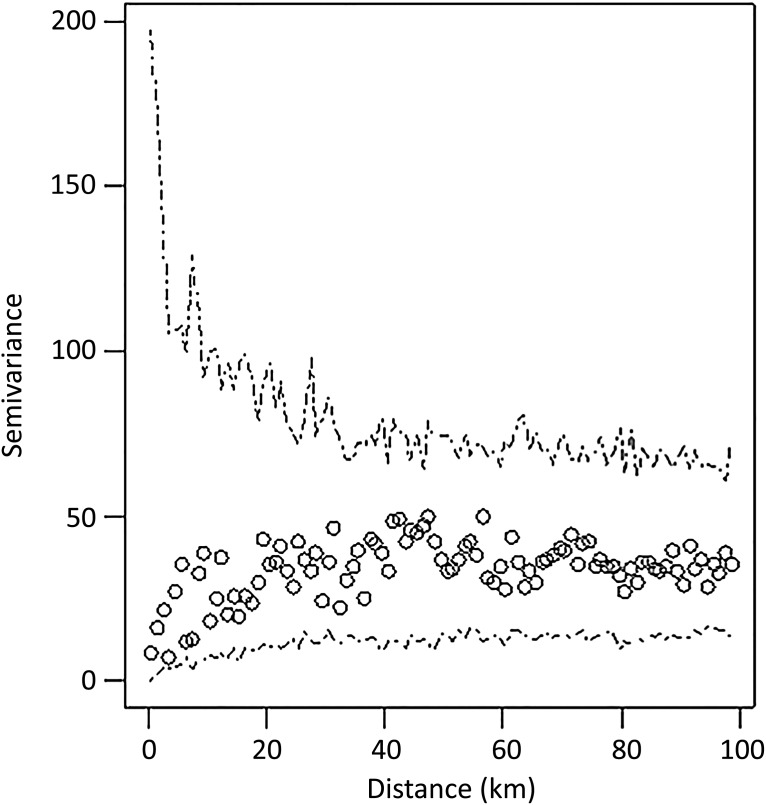
Variogram quantifying the semivariance between pairs of observations (herd residuals from the logistic regression model) as the function of their Euclidean distance. The envelopes were based on 999 Monte Carlo permutations of the data, whereby positive and negative herds were randomly allocated to each farm location. In all, 1537 Swedish dairy herds tested in 2008–2009 were included in this study on risk factors for *Coxiella burnetii* infection.

Finally, as shown in Figure 5, the residuals of the model were distributed within the envelope. Thus, the choice of a classic logistic regression instead of a spatial model accounting for the spatial autocorrelation was appropriate.

## DISCUSSION

Spatial analysis identified areas in the south of Sweden associated with higher risk of infection. Factors that could explain this spatial pattern were investigated. For herds located in areas with high wind speed, open landscape, high animal densities and high temperature, the risk of being infected reached very high values, up to 42. Finally, cumulated precipitation was identified as a protective factor, i.e. associated with a decreased risk of infection.

This study was conducted at the national level and the high number of herds included in the study allowed a high statistical power to be reached. Moreover, a random selection was applied on a sampling frame covering more than 95% of the Swedish dairy herds. Indeed, as showed in Figure 3, herds were selected from all the different regions, which indicated a good representativeness of the data. The strategy used to quantify the contributions of the different factors was a multivariable approach that allowed (i) controlling for known factors that influence the probability of infection and (ii) ranking the impacts of the factors of interest.

Results of this study indicated that wind speed is a major risk factor of infection. Several studies are supporting this finding both in ruminants and between ruminants and humans [10–12, 34]. Differences in the magnitude of effects between crude and adjusted associations highlight the interest in considering adjustment variables.

Unadjusted associations show a greater effect of cattle density compared to sheep density on the risk of being ELISA positive. This result is in accordance with a recent study describing prevalences of *Cb* antibodies within cattle, sheep and goat populations in Sweden. The overall prevalence of *Cb* antibodies was 8·2%, 0·6% and 0% in cattle, sheep and goat populations, respectively [19]. The effect of sheep density on the risk of infection found in the present study reflects the strong spatial correlation between these two populations (Fig. 1). Sweden has experienced a very small number of Q fever human cases in recent years [35], this may be related to the absence (or quasi-absence) of *Cb* in small ruminant populations. Indeed, small ruminants are identified as the source in the majority of outbreaks in humans [36].

It was found that an increase in cumulative precipitation was associated with a decreased risk of infection. To our knowledge, this is the first analytical epidemiological study that demonstrates this effect on the risk of *Cb* infection in animals. Precipitation could decrease the quantity of bacteria aerosolized, which is a main determinant of its infectiousness. This finding demonstrates what was previously described in studies investigating environmental risk factors for Q fever infection in humans. One study has shown that lower rainfall in the previous year was associated with an unusual peak of Q fever human infections related to the presence of sheep within the neighbourhood [11]. In northern Queensland (Australia), the greatest number of human cases was observed at the beginning of the dry season (3 months after the peak in rainfall) [37]. The authors hypothesized that both an increase in wildlife numbers and drier conditions following the wet season could explain the seasonal peak of human acute Q fever cases. It has also been demonstrated that the soil moisture decreased the risk of Q fever infection by reducing the amount of dust available for dispersion of the bacteria [38].

The reported results suggest that the most relevant control measure to be implemented to limit the spread of infection could vary as a function of the environmental and climatic factors (wind speed, type of landscape, animal densities and temperature) in the area. Indeed, these factors – that are difficult to control – had a greater contribution to the risk of infection compared to animal movements. In herds located in areas with a high exposure (open windy areas with high animal densities and high temperature), vaccination could be an appropriate control measure in both *Cb*-free and infected herds. Indeed, vaccination of dairy cows prevents the risk of becoming a shedder in animals still *Cb*-free, even in infected herds [9]. By contrast, in areas with low exposure to environmental and climatic factors, measures based on control of animal movements, by trade restriction or control testing, could be sufficient.

The infection status of *Cb* was assessed using a serological test. A positive result could thus either correspond to a current or a past infection. Nevertheless, 453 of the samples used here were concomitantly analysed for detection of the agent using polymerase chain reaction (PCR) [19]. These authors reported a good overall agreement between ELISA and PCR results with 85% of the samples that were positive by both tests. To limit the remaining possible misclassification of cases when assessing the risk of transmission, further studies, preferably longitudinal, could be conducted based on direct detection of *Cb* using PCR. That would allow detecting newly infected herds and thus describing the evolution of herd status over time. Moreover, it would allow better reflection of the time sequence of events.

The meteorological data were aggregated to reflect the year before the determination of herd status. This approach was used because of the uncertainty concerning the date of infection in the context of the present cross-sectional study. Aggregated data over a long period do not allow accounting for temporal variations which could have increased the accuracy of the analysis. Furthermore, there are other variables that could have helped explain the *Cb* herd statuses such as relative humidity, soil moisture and the type of vegetation. However, cumulative precipitation partially accounts for relative humidity and soil moisture. Regarding vegetation, the type of landscape only accounts for the fact that land with vegetation is not considered as open. A study using more comprehensive weather and environmental data could be considered.

Meteorological factors are likely to play similar roles on the risk of infection in different contexts. In other words, it can be assumed that wind speed would still be a major risk of infection and precipitation a protective factor in other regions. However, their relative contributions on the risk of infection may differ in relation to possible differences in the farming systems (e.g. animal density, species, level of animal movement) and the type of landscapes under study.

### Conclusion and practice implications

The factors influencing the transmission of the bacteria between ruminant herds should also impact the transmission between herds and humans. Therefore, the present findings concerning risk factors of infection in dairy cattle herds could contribute to a better prevention of infection in humans, especially for those who live in peri-urban areas. Especially, in windy areas with open landscape, high animal densities and high temperatures, the vaccination of cattle in both infected and *Cb*-free herds may be a relevant control measure to limit zoonotic risk. Finally, cumulative precipitation was identified as a protective factor which is, to our knowledge, the first analytical epidemiological study that demonstrates this effect.
